# Brief report: joint trajectories of anxiety and depression symptoms in an inception cohort of autistic youth

**DOI:** 10.3389/fpsyt.2026.1741956

**Published:** 2026-05-05

**Authors:** Michelle C. Hunsche, Anat Zaidman-Zait, Melissa Olana, Peter Szatmari, Teresa Bennett, Eric Duku, Stelios Georgiades, Isabel M. Smith, Lonnie Zwaigenbaum, Mayada Elsabbagh, Tracy Vaillancourt, Rachael Bedford, Connor M. Kerns

**Affiliations:** 1 Department of Psychology, University of British Columbia, Vancouver, BC, Canada; 2 School of Education, Tel Aviv University, Tel Aviv, Israel; 3 Department of Psychiatry, University of Toronto, Toronto, ON, Canada; 4 Offord Centre for Child Studies, Department of Psychiatry and Behavioural Neurosciences, McMaster University, Hamilton, ON, Canada; 5 Department of Pediatrics, Dalhousie University, Halifax, NS, Canada; 6 Department of Pediatrics, University of Alberta, Edmonton, AB, Canada; 7 Department of Neurology and Neurosurgery, McGill University, Montreal, QC, Canada; 8 Counselling Psychology, Faculty of Education, University of Ottawa, Ottawa, ON, Canada; 9 Centre for Brain and Behaviour, Department of Psychology, Queen Mary University of London, London, United Kingdom

**Keywords:** adolescence, anxiety, autism, childhood, depression, longitudinal trajectories

## Abstract

**Background and Aims:**

Anxiety and depression symptoms are common among autistic youth, yet little is known about the pattern and relationship of their trajectories from childhood into adolescence, a period of increasing social and academic demands.

**Methods:**

This study used parallel process latent growth curve models to examine joint trajectories, including initial levels and rate of change in caregiver-reported depression and anxiety symptoms across age 7–16 within an inception cohort of autistic youth with varied communication abilities. We also examined autistic traits, sex assigned at birth, emotional reactivity and communication ability as potential predictors. Child anxiety and depression symptoms were estimated from Child Behavior Checklist Anxiety and Affective Problems subscales, completed by caregivers approximately annually.

**Results:**

Whereas anxiety symptoms were relatively stable from childhood into adolescence, depression symptoms increased on average; significant heterogeneity of individual trajectories underlaid these overall trends. Findings indicated cross-sectional and longitudinal co-occurrence of anxiety and depression symptoms. Greater autistic traits and emotional reactivity correlated with greater initial anxiety and depression symptoms, but not their trajectories. Stronger communication ability correlated with more initial anxiety, but decreasing anxiety symptoms over time.

**Conclusions:**

Findings indicate group-level changes in depression symptoms and synchronous evolution of anxiety and depression symptoms in autistic youth across childhood and adolescence. This indicates the importance of joint monitoring of anxiety and depression symptoms in this period, with changes being potentially informative for early detection and intervention. Considering how anxiety symptom presentation may evolve across development may be a helpful next step to identifying at-risk subgroups.

## Introduction

Autism spectrum disorder, hereafter “autism,” is a neurodevelopmental condition diagnosed in approximately 1 in 100 children worldwide (1) and characterized by social communication differences and repetitive patterns of behavior and interest (2). Relative to allistic (i.e., non-autistic) youth, autistic^1^ youth are more likely to be diagnosed with anxiety disorders (39.5% v. 6.3%) and depression (15.7% v. 2.8%) (3, 4). Recent longitudinal work has identified the school-aged and adolescent years as periods of increasing internalizing concerns (e.g., anxiety, depression) for autistic youth (5–7). In a population-based study involving autistic and allistic youth followed across age 4, 7 and 13 (*n* = 135), the most common age of onset of internalizing difficulties was 7 years, and autistic youth were more likely to demonstrate persistent internalizing concerns across childhood and early adolescence (5). Similarly, Wright et al. (2023) reported consistently elevated internalizing symptoms in autistic compared to allistic ages 2 to 10 in the same sample as the current study (Pathways in ASD) (6). In a study following internalizing problems in autistic and allistic youth from ages 13 to 24 years, anxiety and depression symptoms began and remained higher in the autistic group, with autistic girls demonstrating increasing symptoms through adolescence and autistic boys demonstrating stable high symptoms (7).

Evidence also suggests an interactive developmental course of anxiety and depression in allistic children, such that the presence of an anxiety disorder may predict the emergence of depression symptoms and vice versa (8–10). In autistic youth, studies suggest anxiety symptoms in late childhood or early adolescence may predict depression symptoms in late adolescence or young adulthood, but depression symptoms have not predicted later anxiety (11–13). Qualitative studies with autistic youth and adults also highlight how anxiety and depression symptoms can contribute to social withdrawal, occupational challenges, and loneliness, which in turn exacerbate mental health challenges (14, 15). However, most trajectory studies involving autistic samples have used broad measures of internalizing symptoms, and no studies to date have examined the progression and association of anxiety and depression symptoms across development – that is, the joint trajectories of these symptoms – in autistic youth. Understanding how anxiety and depression symptoms change and interact over time may be particularly important during the transition to adolescence, a time of heightened social and environmental demands as well as increased risk of some internalizing problems (16, 17). Investigating these trajectories together may illuminate their relationship and inform efforts to identify and treat internalizing concerns early.

Understanding factors associated with the onset and maintenance of anxiety and depression symptoms for autistic youth is also an active area of inquiry (18). Evidence from cross-sectional and longitudinal research indicates that restricted and repetitive behavior is associated with greater anxiety symptoms among autistic individuals (19–21). Social communication differences may also indirectly predispose autistic youth to internalizing problems through contributing to challenges in developing and maintaining friendships and by increasing risk of negative social outcomes such as bullying (22, 23). Conversely, stronger language and communication skills have been linked to greater anxiety symptoms in autistic children, though findings are mixed (3, 24–27). Autistic people also experience notably more negative affect, greater difficulty coping with distress, and greater emotional reactivity than allistic people across the lifespan (28, 29). Researchers have argued that increased emotional reactivity experienced by autistic people may partially explain the high rates of co-occurring mood, anxiety, and related outcomes (e.g., suicide-related outcomes) among this population (30, 31). Finally, as in allistic youth, sex assigned at birth has been linked to differences in the development of internalizing problems in autistic youth, with autistic females demonstrating elevated depression and anxiety symptoms compared to autistic males and allistic females in some studies (32, 33). Clarifying whether these risk factors are similarly or distinctly related to anxiety and depression trajectories specifically (rather than to internalizing symptoms generally, as in prior work (5, 6)) could inform early intervention and prevention efforts and help identify children most at risk.

Against this backdrop, we sought to (1) examine joint trajectories of caregiver-reported anxiety and depression symptoms, including associations between initial levels and rate of change in depression and anxiety symptoms, across late childhood and early adolescence (ages 7-16) within an inception cohort of autistic youth, and (2) test potential shared vs. distinct predictors (i.e., emotional reactivity, sex assigned at birth, communication ability, level of autistic traits) of these joint symptom trajectories.

## Methods

### Participants and procedure

This study involved select data from *Pathways in ASD*, a longitudinal inception cohort study examining developmental trajectories of autistic children across five Canadian provinces (Nova Scotia, Quebec, Ontario, Alberta, British Columbia). The initial sample assessed at Time 1 (*N* = 421; 2–4 years old) was recruited starting in 2005 upon receiving a recent (within four months) autism diagnosis at regional autism assessment centers. Diagnoses were made by clinicians with autism expertise using DSM-IV-TR criteria (34) and both the Autism Diagnostic Observation Scale (ADOS) (35) and Autism Diagnostic Inventory-Revised (ADI-R) (36). To be included in the study, caregivers also had to be proficient in English (or French if in Quebec). Participants were excluded at study intake if they had a prior diagnosis of a neuromotor disorder (e.g., cerebral palsy), genetic/chromosomal abnormalities, or significant vision/hearing impairments. Caregivers provided informed consent before data collection. Participants were assessed across three phases of development: Phase I, early childhood (age of diagnosis to 6 years old; Time 1 to Time 4); Phase II, school-age (7 to 11 years old; Time 5 to Time 8); and Phase III, adolescence to emerging adulthood (12–19 years old, Time 9 to Time 11 [Time 12 data collection ongoing]). For this study, we focused on data from Phase II, Time 5 to Time 8 (7 to 12 years old) and Phase III, Time 9 (12–15 years old). Phase II and III assessments involved a comprehensive battery of cognitive, social and behavioral measures. Participants were included in the current study if caregivers had completed the key measure of interest, the Child Behavior Checklist (CBCL) (37), at one or more time points from Time 5 to Time 9 (7 to 14 years old). The final sample included 277 autistic children. We found no significant differences in household income, sex, or ethnicity in those who completed the CBCL at one or more time points (final sample; *n* = 277) and those who did not (*n* = 144). There are also no group differences in Time 4 ADOS Severity Scores or Social Responsiveness Scale (SRS-2 (38);) total scores in those included versus excluded from analyses.

### Measures

#### Trajectory variables

##### Achenbach System of Empirically Based Assessment Scales Child Behavior Checklist

The CBCL for ages 6–18 (37) is a caregiver-report measure of emotional and behavioral problems in children in the past 6 months. It contains subscales consistent with internalizing disorders per the Diagnostic and Statistical Manual of Mental Disorders-Fourth Edition (DSM-IV; 34), including Anxiety Problems and Affective Problems. These DSM scales show internal consistency (α <.70) among autistic children and adolescents (39). The Affective measure has also demonstrated excellent sensitivity (0.95 AUROC) across three population-based datasets of youth (40). We used total raw scores on these subscales at Times 5–9 to assess child anxiety and depression symptoms, respectively.

#### Predictor variables

##### Social Responsiveness Scale

The SRS (38) is a continuous measure of caregiver-reported autistic traits that was tested as a predictor of anxiety and depression trajectories and used to characterize the sample. It measures autistic traits as continuous, dimensional characteristics that are present in the general population. The SRS has demonstrated sound psychometric properties among autistic youth, including good internal consistency (.93), inter-rater (.75-.91), and test-retest reliability (.83-.88) (41, 42).

##### Behavior Rating Inventory of Executive Function

The BRIEF (43) is a caregiver-reported measure of executive functioning-related behavior in day-to-day life. It is argued to have greater ecological validity than performance-based executive functioning measures (44) and shows convergent validity on informant-rating and performance measures and high test-retest reliability (45). The Emotional Control subscale, which assesses executive function within the context of emotional experiences (hereon referred to as emotional reactivity), was tested as a predictor of anxiety and depression trajectories.

##### Vineland Adaptive Behavior Scale, 2^nd^ Edition

The VABS-II (46) is a semi-structured interview assessing communication, daily living, socialization, and motor skills. The VABS has demonstrated good content and convergent validity and internal consistency in autistic youth (46–48). Higher scores correspond to better adaptive functioning. The VABS Communication domain total was tested as a predictor of anxiety and depression trajectories.

### Data analytic plan

To examine the parallel developmental processes of anxiety and depression symptoms from late childhood into early adolescence, we applied a parallel process latent growth curve modeling (PP-LGCM) approach. PP-LGCM simultaneously estimates growth trajectories of multiple constructs capturing both individual differences in initial levels (intercepts) and rates of change (slopes) over time (49). In addition, PP-LGCM allows estimation of the associations between growth processes across domains.

**Figure 1:**
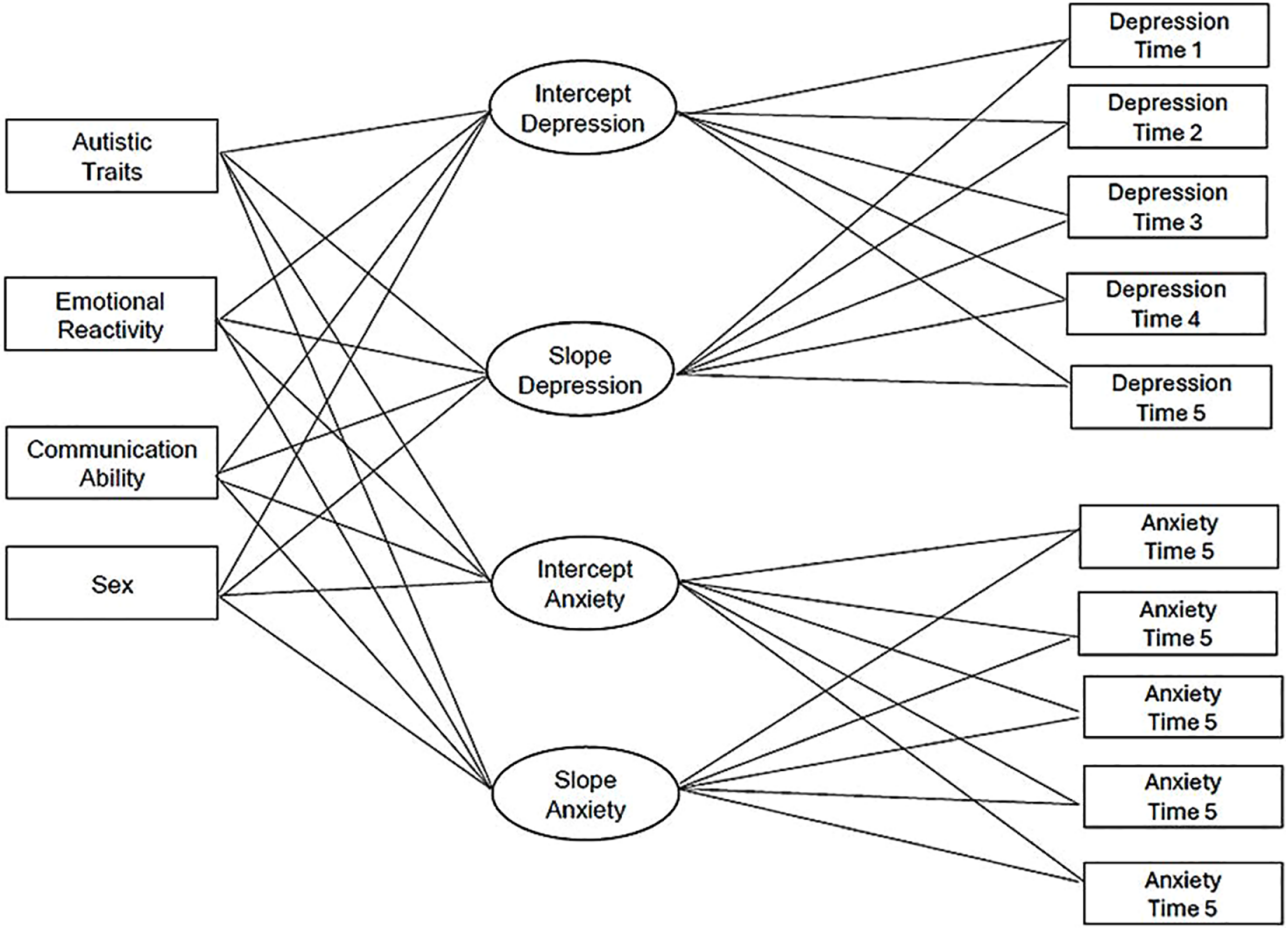
Conceptual model of anxiety and depression symptom trajectories and predictors.

In the first step, we specified an unconditional PP-LGCM (49) using mean scores of anxiety and depression symptoms across five waves of assessment (see **Figure 1** for a conceptual model). The model accounted for unequal spacing at the final wave to reflect the longer interval between the last two assessments. The intercept was centered at the first wave (i.e., age 7), and the slope represented the annual rate of change. To account for the extended interval between the fourth and fifth assessment, slope factor loadings were fixed to reflect actual years elapsed. This approach ensures that the estimated rate of change reflects the actual temporal spacing of observations (50). To determine the shapes of anxiety and depression symptom trajectories, we included a linear slope and then a quadratic slope, using fit statistics to determine the best trajectory model. Residual variances were allowed to be freely estimated to accommodate developmental changes in measurement precision while maintaining construct validity (51). In the second step, we estimated a conditional PP-LGCM in which children’s emotional reactivity, autistic traits, sex assigned at birth, and communication ability at age 7 were specified as time-invariant predictors of the growth factors (intercepts and slopes) for anxiety and depression. In addition, children’s family income was included as covariates to account for demographic variability in symptom trajectories.

Model fit was evaluated based on convergence across multiple indices, including the root mean square error of approximation (RMSEA), standardized root mean square residual (SRMR), and comparative fit index (CFI). Excellent model fit was defined as RMSEA <.06, SRMR <.08, and CFI >.95, and an acceptable fit was defined RMSEA <.08 and CFI >.90 (52). We used convergence across indices rather than relying on a single criterion because different fit indices are sensitive to different aspects of model misspecification. Evaluating multiple indices therefore provides a more comprehensive assessment of model adequacy (53). All PP-LGCMs were estimated in Mplus version 8.9 (54) using full-information maximum likelihood (FIML) to handle missing data and maximum likelihood estimation with robust standard errors (MLR) to account for non-normality. Significance of findings was determined by reaching the conventional significance threshold *(p* <.05). Preliminary descriptive analyses were conducted in SPSS version 29.0.

## Results

### Participant characteristics

Descriptive statistics of the sample for study variables are reported in **Table 1**. Most participants were White (74.3%) and male (84.5%). Average sample IQ was in the average to low average range (approximately 19% with full-scale IQ < 70), and most (77%) scored at or above a standard score of 70 on the VABS Communication domain (mean of 100, standard deviation of 15). Of 204 participants who completed the ADOS at Time 8, 74% met the total score cut-off for autism/autism spectrum (all received a community diagnosis of autism spectrum disorder at study onset at T1).

**Table 1:** Sample sociodemographic and clinical characteristics.

Variable	Full sample (N = 277)		
	Available N	M(SD) or n(%)	Min-Max
Sex at Birth	277		
Male		234 (84.5%)	
Female		43 (15.5%)	
Ethnicity	265		
White		197 (74.3%)	
East Asian		17 (6.4%)	
Arab/West Asian		10 (3.8%)	
Black		8 (3.0%)	
Indigenous		3 (1.1%)	
South & Southeast Asian		19 (7.2%)	
Latin		6 (2.3%)	
Other		5 (1.9%)	
Household Income (CAD)	258		
<$20,000		23 (8.9%)	
$20,000 - $39,999		33 (12.8%)	
$40,00 - $59,999		50 (19.4%)	
>$60,000		152 (58.9%)	
Site	277		
Edmonton, AB		37 (13.4%)	
Halifax, NS		40 (14.4%)	
Hamilton, ON		32 (11.6%)	
Montreal, QC		106 (38.3%)	
Vancouver, BC		62 (22.4%)	
Full scale IQ^a^	175	84.8(18.7)	41-131
CBCL: T5 Affective Problems T Score	197	57.6 (7.4)	50-79
CBCL: T5 Anxiety Problems T Score	197	58.1(7.7)	50-77
SRS Parent T5 T Score	198	73.8(16.4)	37-112
VABS Parent T4 Communication Standard Score	246	81.4(15.7)	48-130
BRIEF Emotional Control T Score	197	56.7(12.3)	35-85

BRIEF, Behavior Rating Inventory of Executive Functioning; CAD, Canadian dollars; CBCL, Child Behavior Checklist; SRS, Social Responsiveness Scale-2^nd^ edition; VABS: Vineland Adaptive Behavior Scales, 2^nd^ edition; WISC-IV, Wechsler Intelligence Scale for Children - Fourth Edition.

#### Unconditional parallel process - latent growth curve model

The quadratic model showed estimation problems and non-significant quadratic slope parameters. Specifically, the quadratic model terminated normally but produced a non-positive definite information matrix, preventing computation of standard errors for the quadratic slope variance. This indicates the data structure did not provide sufficient information to reliably estimate the quadratic component. Accordingly, linear growth was retained. The unconditional PP-LGCM demonstrated excellent fit to the data, χ²(36) = 41.97, *p* = .23, RMSEA = .02 (90% CI [.000,.051]), CFI = .99, TLI = .99, SRMR = .04). Average trajectories revealed that while anxiety symptoms remained stable (slope *M* = 0.01, *SE* = 0.01, *p* = .34), depression symptoms showed a significant increase (slope *M* = 0.01, *SE* = 0.003, *p* <.001). Intercept means were significant for both anxiety (*M* = 0.47, *SE* = 0.03, *p* <.001) and depression (*M* = 0.20, *SE* = 0.01, *p* <.001). Significant individual differences were seen in both initial levels and rates of change for anxiety (intercept: σ² = 0.13, *p* <.001; slope: σ² = 0.003, *p* = .01) and depression symptoms (intercept: σ² = 0.03, *p* <.001; slope: σ² = 0.001, *p* = .01), indicating heterogeneous developmental trajectories.

Anxiety and depression intercepts were moderately positively correlated (*r* = .54, *p* <.001), indicating initial co-occurrence. Slopes were also moderately positively correlated (*r* = .53, *p* <.01), indicating synchronized development. In other words, youth with steeper change in one domain, whether increasing or decreasing, tended to show steeper change in the other domain in the same direction. Within-domain associations between initial levels and slopes were not significant for either anxiety (*r* = -.19, *p* = .12) or depression (*r* = -.09, *p* = .52), indicating that initial symptom severity did not predict individual trajectories within each construct. Cross-domain associations were also not significant (anxiety intercept with depression slope: *r* = -.04, *p* = .79; depression intercept with anxiety slope: *r* = .08, *p* = .58).

#### Conditional parallel process - latent growth curve model

The conditional model included predictors (communication ability, emotional reactivity, autistic traits) to examine factors associated with initial symptom levels and change. Sex assigned at birth was included as a predictor of initial levels only, as preliminary models indicated sex did not predict change trajectories. Specifically, sex was not significantly associated with initial levels of anxiety (β = -0.089, *SE* = 0.056, *p* = .115) or initial levels of depression (β = 0.010, *SE* = 0.029, *p* = .722). Additionally, sex did not significantly predict change in anxiety symptoms over time (*b* = 0.002, *SE* = 0.016, *p* = .892) or change in depressive symptoms (β = -0.007, *SE* = 0.010, *p* = .468). The conditional model demonstrated good fit, χ²(62) = 74.38, *p* = .13, RMSEA = .03, 90% CI [.00,.05], CFI = .99, TLI = .99, SRMR = .04.

**Figure 2:**
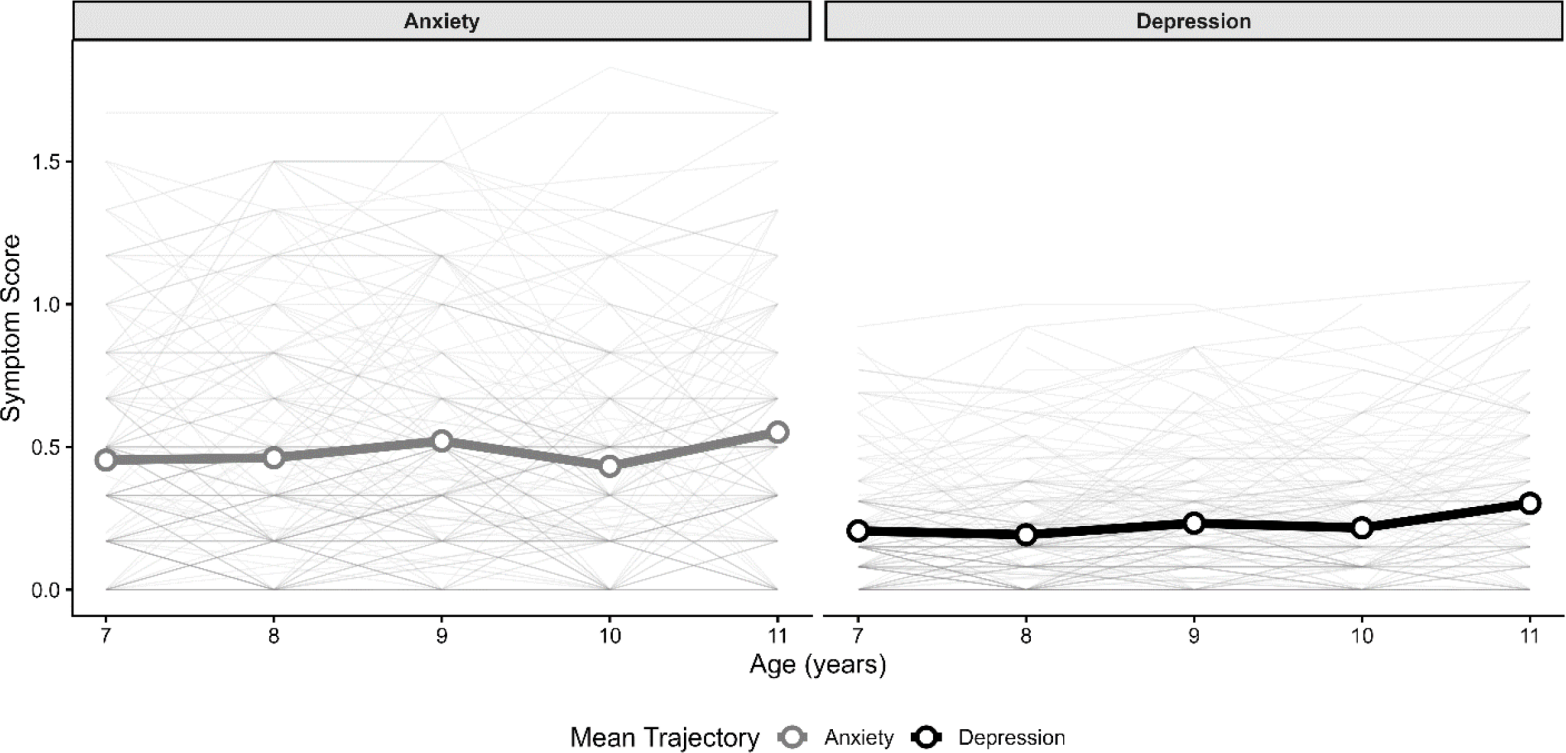
Individual and mean trajectories of anxiety and depressive symptoms. Individual trajectories are shown in gray; mean trajectories are shown in bold.

Consistent with the unconditional model, the mean slope of anxiety was non-significant (*M* = .003, *p* = .65), whereas the mean slope of depression was significant (*M* = .01, *p* <.001), indicating gradual increases in depression symptoms from late childhood to adolescence. Significant individual variability also persisted in both intercepts (anxiety σ² = .07, *p* <.001; depression σ² = .02, *p* <.001) and slopes (anxiety σ² = .002, *p* = .01; depression σ² = .001, *p* = .003). In addition, initial anxiety and depression levels remained moderately correlated (*r* = .23, *p* = .02), and individual change trajectories remained strongly correlated (r = .59, *p* = .003). See **Figure 2** for a spaghetti plot of anxiety and depression symptom trajectories.

Higher levels of emotional reactivity per the BRIEF Emotion Control subscale (anxiety β = .21, *p* = .004; depression β = .20, *p* = .01) and autistic traits per the SRS (anxiety β = .59, *p* <.001; depression β = .58, *p* <.001) was associated with higher initial levels of anxiety and depression symptoms. Higher communication scores were associated with higher initial anxiety symptoms (β = .33, *p* <.001), but also steeper anxiety declines over time (β = -.27, *p* = .05).

Communication ability per the VABS was not associated with either initial level (β = .004, *p* = .96) or rate of change in depression symptoms (β = .14, *p* = .31). Emotional reactivity (anxiety β = -.19, *p* = .18; depression: β = -.03, *p* = .82) and autistic traits (anxiety β = -.24, *p* = .15; depression β = -.15, *p* = .38) was not associated with either anxiety or depression change. Finally, sex assigned at birth did not significantly predict initial levels of either anxiety (β = -.09, *p* = .19) or depression (β = .003, *p* = .96). Predictors explained substantial variance in initial symptom levels (anxiety: *R²* = .41; depression: *R²* = .51) but minimal variance in trajectory slopes (anxiety: *R²* = .13, *p* = .13; depression: *R²* = .07, *p* = .24).

## Discussion

We examined joint trajectories of caregiver-reported anxiety and depression symptoms from the school-age to adolescent years in an inception cohort of autistic youth with a wide range of communication abilities who were diagnosed in early childhood. In the current sample, depression symptoms increased from childhood into adolescence, though modestly, while anxiety symptoms remained stable. Anxiety and depression symptoms were cross-sectionally associated at age 7 and symptom trajectories were positively correlated across development, indicating synchronized individual variability despite different average trends. Greater autistic traits and emotional reactivity at age 7 were associated with greater anxiety and depression cross-sectionally, but did not predict symptom change over time. Finally, higher communication abilities at age 7 were unrelated to depression, but predicted more initial anxiety yet decreasing anxiety symptoms over time. These findings are discussed in turn below.

The mean trajectory of anxiety was stable across ages 7 to 15 years in the current sample, albeit with significant individual variability. These findings are consistent with a prior study, which also found internalizing symptoms remained relatively stable across this developmental period (5). Our findings further suggest that this stability may particularly apply to anxiety symptoms, which other research shows may arise early in childhood for autistic youth (preschool period), but then plateau on average in severity from childhood into adolescence (11, 55–58). Whether the expression of these anxiety symptoms is as homotypic across development is less clear. Specifically, symptoms of anxiety disorders more common in early childhood (e.g., separation anxiety, specific phobia) may evolve into generalized and/or social fears in middle childhood and early adolescence, as observed in allistic youth (59, 60). Thus, consistent with Weems’ developmental model of continuity and change of anxiety symptoms (61), the focus or source of anxiety may shift as developmental demands change, despite relatively stable mean trends in the number and intensity of symptoms. Moreover, consistent with the significant variability in individual anxiety symptom trajectories we observed, trajectories of anxiety symptoms are likely heterogenous despite the appearance of group-level stability. For example, in the Pathways in ASD sample, Baribeau and colleagues (62) identified four distinct anxiety symptom trajectories across ages 3 to 10 among autistic children, including a high-stable and high-increasing group. It may be that internalizing symptoms remain stable for most autistic youth, while a subset experience increasing or decreasing trajectories; group-based trajectory modeling methods could help clarify predictors of such anxiety symptom trajectory groups for autistic adolescents and young adults in future research. It is also possible that internalizing symptom trajectories may follow a non-linear pattern, particularly in adolescence when symptoms may increase in prevalence and severity (7, 12), though this could not be explored in the current study due to estimation problems in the quadratic model. These findings highlight the importance of monitoring changes in autistic youth’s anxiety over time to identify individuals with emerging or accelerating risks and intervene early.

Depression symptoms increased on average, albeit modestly, a pattern not apparent in a prior study that examined the trajectory of internalizing problems collectively in autistic children (5). These findings replicate prior work linking adolescence and depression in both allistic (63, 64) and autistic samples (7, 11, 65) and suggest a need for routine monitoring of these symptoms during this period. Additionally, trajectory studies following autistic youth into adulthood suggest that the severity of depression symptoms may continue to increase in later adolescence and peak in early adulthood (age 20) (12), further highlighting the importance of identifying and addressing mood concerns before they become more severe.

Although average anxiety symptoms remained stable while depression symptoms increased, the significant correlation between individual trajectories indicates potentially interconnected developmental processes. Children who showed steeper changes in anxiety, either increasing or decreasing, also tended to show steeper changes in depression (though directionality of this relationship cannot be established by PP-LGCM). Causal mechanisms cannot be determined and may reflect a variety of factors, such as shared underlying mechanisms (e.g., genetic vulnerability to internalizing symptoms) and measurement covariation (e.g., shared variance due to informant bias or use of subscales from the same measure). However, most predictors (including sex) did not explain significant change in internalizing symptoms. Although emotional reactivity, communication ability and autistic traits at age 7 explained substantial variance in anxiety and depression symptoms cross-sectionally, they accounted for minimal and mostly non-significant variance in symptom change over time. Other contextual, time-varying or developmental factors not captured in the current study – such as social adversity, access to psychosocial interventions, or family or environmental stressors – may also play a role in the development of internalizing symptoms and are worth exploring in future trajectory studies. For example, school transitions and changes in social context (e.g., losing access to welcoming, supportive social environments) may contribute to increasing internalizing symptoms (66). It is possible that the presence of shared, transdiagnostic risk factors may explain the synchronous change in anxiety and depression symptoms over time observed here, though testing this is beyond the scope of the current study.

This pattern of synchronized individual variation, despite different average trajectories, underscores the need to assess anxiety and depression together rather than in isolation. This is an important finding given that efforts to develop empirically supported assessment and treatment approaches for autistic youth have primarily targeted anxiety rather than depression symptoms, despite their intertwined nature (67). Our findings suggest that autistic youth with elevated or escalating anxiety symptoms should also be assessed for depressive symptoms, particularly given our findings that depression symptoms tend to trend upward across these developmental periods. Assessing depression in autistic children with worsening anxiety and vice versa may also be critical in shaping the choice and delivery of interventions, such as transdiagnostic approaches that address both anxiety and mood concerns (e.g., the *Secret Agent Society: Operation Regulation* intervention (68)) or modular protocols that have shown greater efficacy for broad internalizing concerns compared to standard cognitive behavioral therapy (CBT) for anxiety (e.g., *Behavioral Interventions for Anxiety in Children with Autism* (69, 70);. For some youth, exclusively treating anxiety might miss an important part of the clinical picture, as depression symptoms may increase risk of critical issues (e.g., suicidality) requiring stabilization before beginning anxiety-focused intervention. Further, depression has been associated with a poorer response to CBT for allistic youth with social anxiety disorder (71). Untangling the specific relationships among different forms of anxiety and depression symptoms from childhood into adolescence may thus be an important future research direction. Notably, prior work has implicated specific longitudinal relationships between social anxiety and depression in allistic youth (72, 73). However, it is unknown whether these generalize to autistic youth or how other distinct but well-documented forms of anxiety in autistic children (e.g., fears related to change, sensory processing, social confusion (74)) may relate to depression symptoms.

Though parent-reported autistic traits correlated with higher parent-reported anxiety and depression at age 7, they did not predict increasing anxiety or depression over time. This finding is consistent with prior work illustrating cross-sectional associations between autistic characteristics and internalizing problems in childhood, particularly insistence on sameness, restricted and repetitive behavior, and social communication differences (75), but less clear longitudinal relationships (76). We found that autistic traits do not necessarily forecast greater internalizing problems as autistic youth transition to adolescence, though they may be useful predictors in early childhood (21).

This study included autistic children with varied communication abilities, including those with mild to severe communication impairments. Interestingly, in this sample, stronger communication ability at age 7 was associated with more anxiety symptoms at age 7 and also a decrease in anxiety symptoms over time. Prior evidence is mixed regarding the relationship between anxiety and communication ability, suggesting a complex relationship (12, 24). Specifically, while studies of autistic youth with profound communication delays find less anxiety in youth with greater communication challenges, the reverse (more anxiety associated with more communication challenges) has been found for verbally fluent youth. One possible interpretation of these findings is that children with better communication abilities have greater awareness of social difficulties and heightened sensitivity to peer judgment, initially elevating anxiety (18, 22, 77–79). However, these communication abilities may also facilitate adaptive coping, including help-seeking, emotional expression, and social problem-solving, which may support symptom decrease over time. Alternative explanations for this finding, including regression to the mean or measurement artifact, should also be considered. Regardless of the explanation, this finding has important clinical implications indicating strong communication abilities should not be interpreted as protective against anxiety in autistic youth.

### Limitations

Participants were of high socioeconomic status, predominantly male and White. Prior evidence from autistic samples suggests trajectories of anxiety and depression symptoms may differ by sex (6, 7). Sex was unrelated to anxiety and depression symptoms in this study; however, this analysis may have been underpowered (*n* = 43 birth-assigned females). Sex, race/ethnicity, and socioeconomic differences can impact access to services, family/community awareness and attitudes toward mental health, and exposure to discrimination and other risk factors (80, 81). Indeed, rates of internalizing disorders are higher among non-White and lower SES autistic youth (82, 83). Future investigation of internalizing symptom trajectories in more diverse samples of autistic youth is needed to further our understanding of how mental health concerns develop and change over time, as well as optimal points for intervention, for autistic youth of varied identities and backgrounds—not only a narrow subset of the autistic community. In addition, while the Pathways sample includes youth with varied cognitive and language ability, a measure of non-verbal IQ was not available at the time points of interest in the current study, limiting our ability to account for the potential link between more developed cognitive ability and elevated internalizing symptoms as reported in prior work (84). Our sample also only included autistic youth diagnosed in early childhood, whose mental health trajectories may differ from those diagnosed later in life (85). Regarding measurement, though some studies support the CBCL’s accuracy in detecting depression symptoms in autistic youth (40), solely relying on caregiver report carries risks of informant bias and may underestimate the internal experiences of youth (3, 86, 87). Prior work has indicated that discrepancies across self- and parent-reported internalizing symptoms are common among autistic and allistic youth, as youth may be better able to report on their internal cognitive and emotional experience across settings while parents may be more aware of behavioral symptoms (e.g., sleep and diet changes) (88). Further, different forms of anxiety, including autism-related anxieties (e.g., sensory-based fears) or anxiety features (e.g., cognitive rigidity), are not assessed by CBCL, which may reduce its sensitivity in autistic youth (3, 26). Future studies should prioritize multi-informant assessment approaches as well as autism-tailored tools (e.g., Anxiety Diagnostic Interview Schedule-Autism Addendum; Anxiety Scale for Children with ASD), which can assess anxiety subtypes, including autism-related expressions of anxiety (3, 73, 86), to refine our understanding of how anxiety and depression present and evolve in ways that may be specific to this population.

## Data Availability

The data are not publicly available as they contain information that could compromise the privacy of research participants. Requests to access these datasets should be directed to chalum@mcmaster.ca.

## Footnotes

1 Following the recommendation of autistic self-advocates and the preference of most autistic people (see 89, 90), identity-first language (e.g., “autistic person”) as opposed to person-first language (e.g., “person with autism”) is used throughout this paper.
